# Bailout technique to rescue the abruptly occluded side branch with collapsed true lumen after main vessel stenting

**DOI:** 10.1007/s12928-015-0376-7

**Published:** 2016-01-11

**Authors:** Atsushi Funatsu, Ryo Hirokawa, Shigeru Nakamura

**Affiliations:** Cardiovascular Center, Kyoto-Katsura Hospital, 17 Yamada-Hirao-cho, Nishikyo-ku Kyoto, 615-8256 Japan

**Keywords:** Bifurcation lesion, Side branch, Percutaneous coronary intervention

## Abstract

Guidewire recrossing into the abruptly occluded side branch (SB) after main vessel (MV) stenting in the coronary bifurcation is difficult, particularly if the SB has a dissection because the true lumen of SB is collapsed by a hematoma and the second guidewire easily goes into the false lumen. This paper reports a bailout technique to rescue the occluded SB that was complicated by a hematoma because of an unsuccessful guidewire recrossing after MV stenting using a small balloon dilation in the collapsed SB true lumen behind the stent strut and wire penetration.

## Introduction

Bifurcation lesion is one of the complex lesions in the percutaneous coronary intervention (PCI) because it is necessary not only to recanalize the main vessel (MV) but also to avoid the side branch (SB) occlusion. SB occlusion has been reported in 6–9 % of bifurcation stenting [1–3]. Therefore, before stent implantation in the MV, a protective guidewire is usually placed into the SB, and after stent implantation, the SB is recrossed by another guidewire from inside of the stent. When the SB is completely occluded, another guidewire must be manipulated to recross the SB only by referring to the jailed guidewire as a marker. However, in some cases, the second guidewire unintentionally enters into subintimal space of the SB, resulting in a dissection and hematoma at the SB. When this occurs, it becomes more difficult to recross into the true lumen of the SB because that is collapsed by the hematoma. If the SB is a small vessel and the patient has no symptoms, it can be left. However, if the SB is a relatively large vessel and the patient has acute chest pain, it might be necessary to reopen the SB.

Here, we report a bailout technique to rescue the occluded right ventricular (RV) branch complicated dissection using a balloon dilation on the first jailed guidewire and penetration by a tapered-tip guidewire (ASAHI Gaia second guidewire; ASAHI Intecc, Japan) from inside the stent strut to pick up an SB true lumen.

## Case

**Fig. 1:**
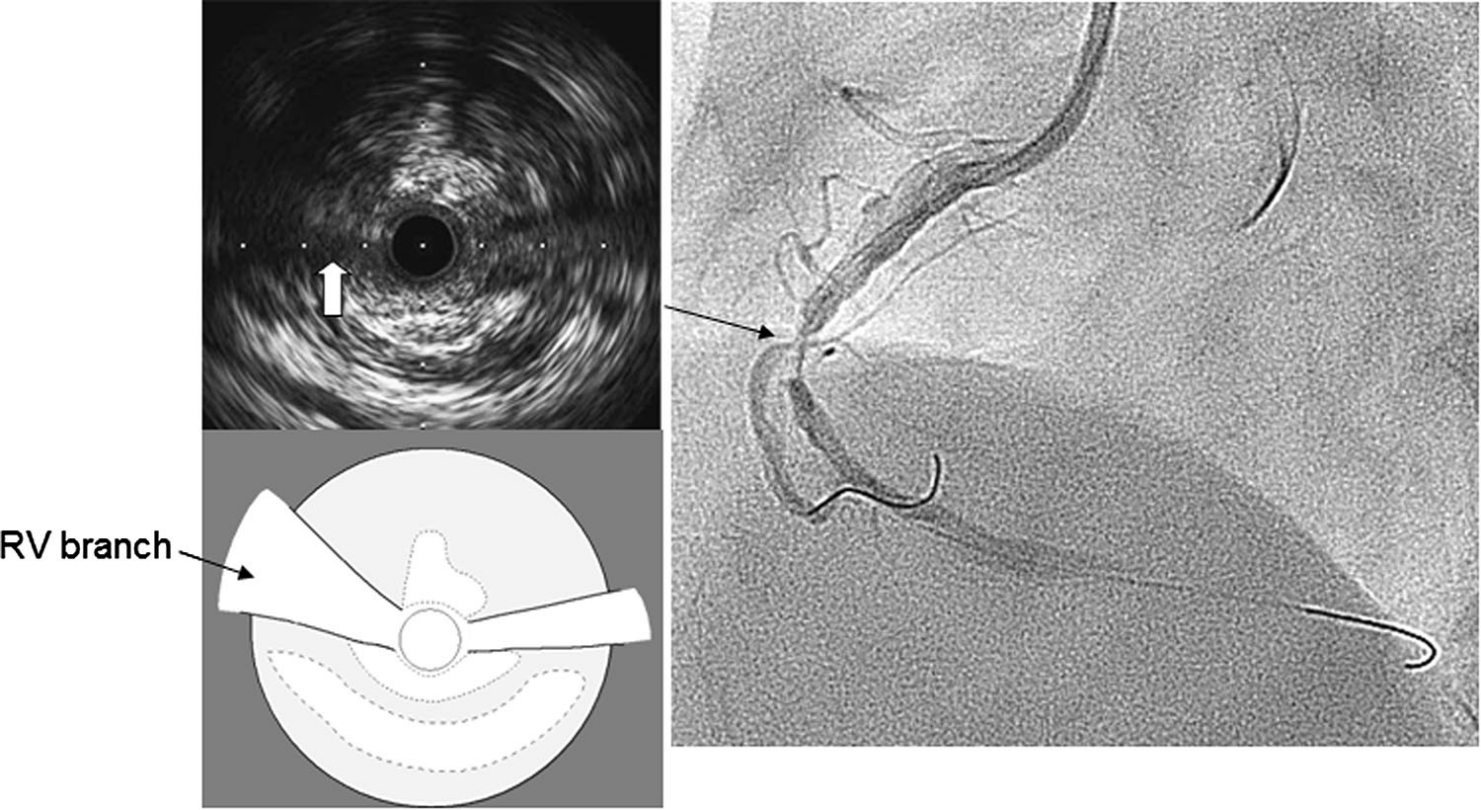
Angiogram and intravascular ultrasound (IVUS) image (Opticross; Boston scientific, Japan) at the right ventricular (RV) branch bifurcation after guidewire crossing and suction. IVUS image revealed that culprit lesion included attenuated plaque, and the RV branch was running through the plaque (*white arrow*). A small branch was also seen at the opposite side of RV branch by the angiogram and IVUS

**Fig. 2:**
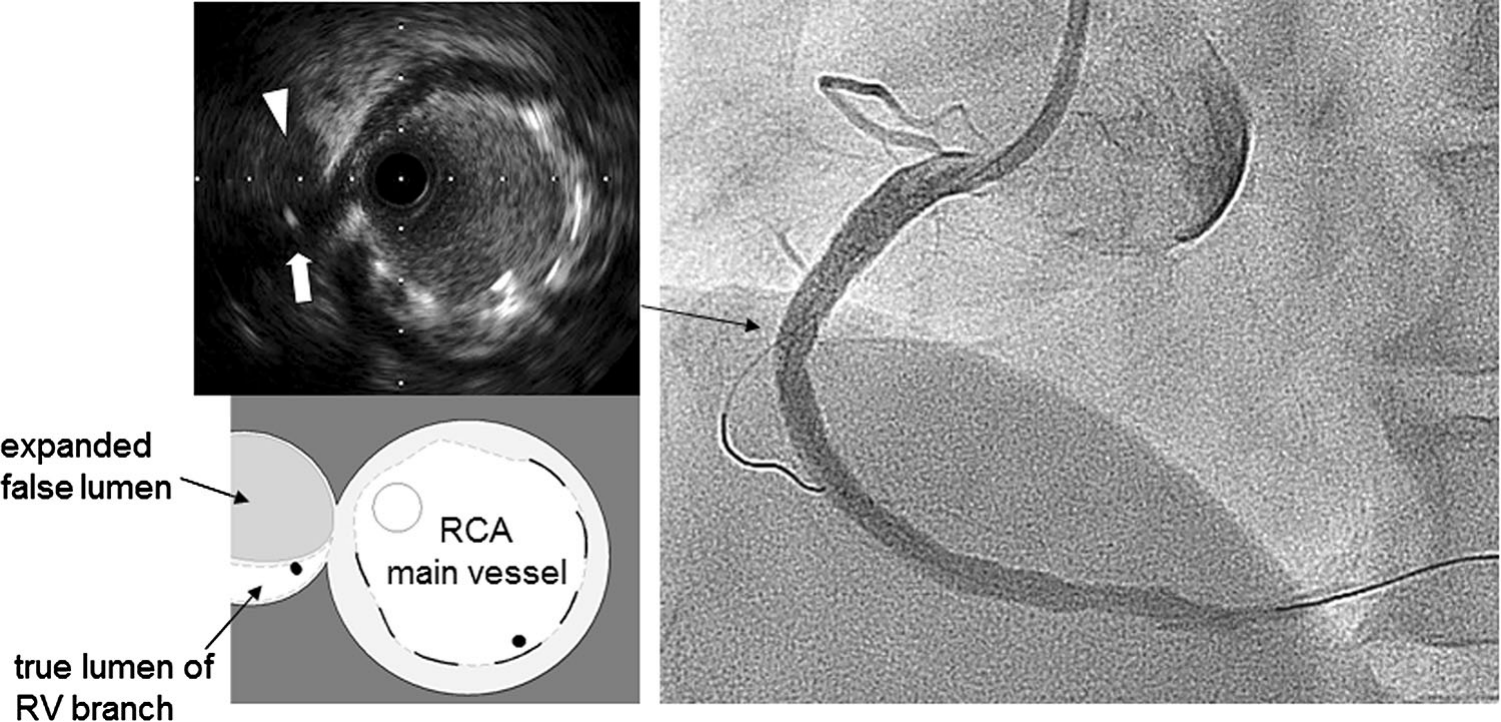
Angiogram after stenting showed that the right ventricular (RV) branch remained completely occluded. Intravascular ultrasound image from the right coronary artery main vessel at the RV branch bifurcation revealed that the RV branch was detected at 9 o’clock position. The true lumen of the RV branch (*white arrow*) was collapsed by an expanded false lumen that was presented as a high echogenic lumen (*white arrowhead*)

**Fig. 3:**
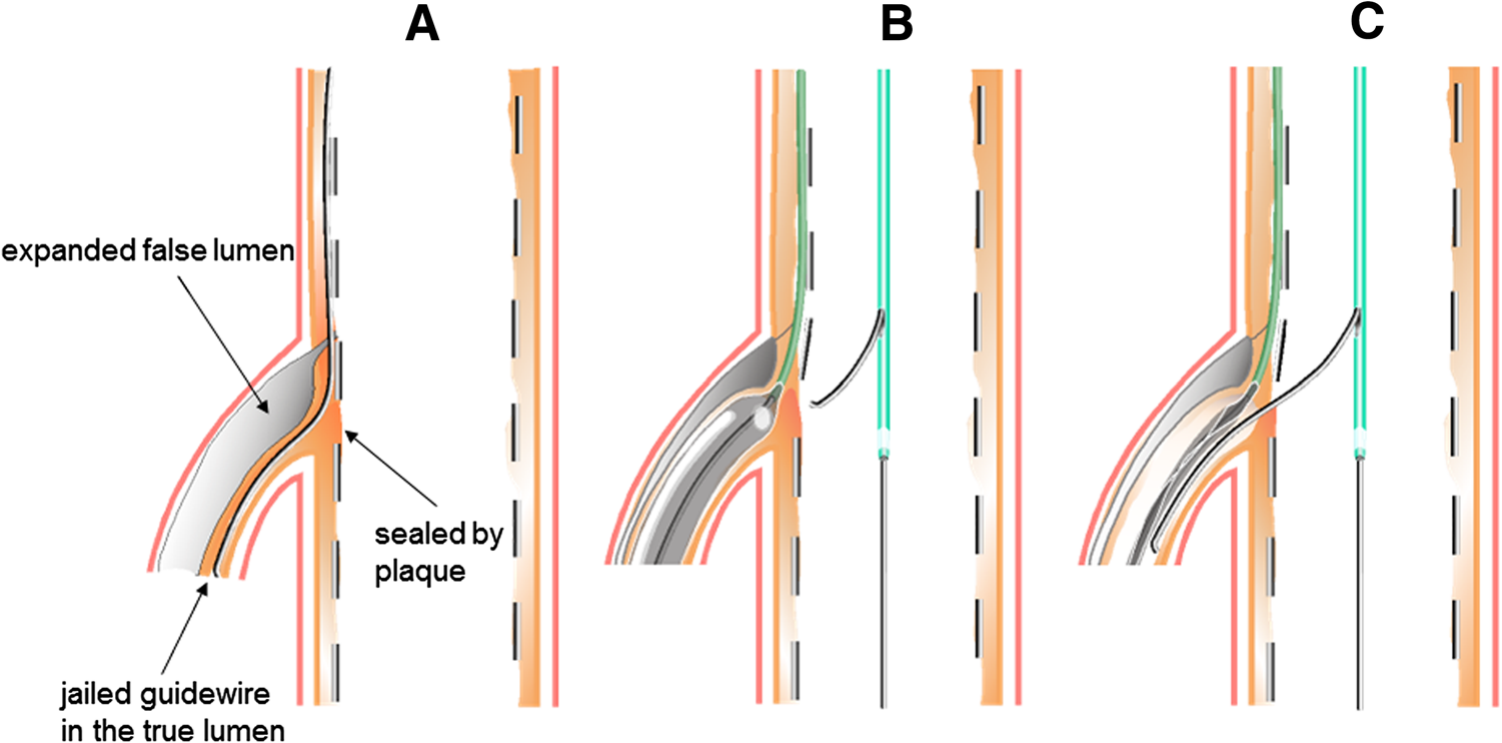
Schematic diagram of how to pick up the right ventricular (RV) branch true lumen. **a** RV branch true lumen, where jailed protection guidewire is existing in, is collapsed by the false lumen. And also the RV branch orifice is sealed by shifted plaque. **b** Aiming the RV branch balloon with chronic total occlusion (CTO) guidewire supported by Crusade catheter to pick up true lumen, while RV branch dilation is in the true lumen using small balloon on the protection guidewire to keep the true lumen open. **c** When CTO guidewire tip is pushing on the balloon through the strut, the balloon is immediately deflated. The guidewire pick up the RV true lumen in the same space of the deflated balloon

**Fig. 4:**
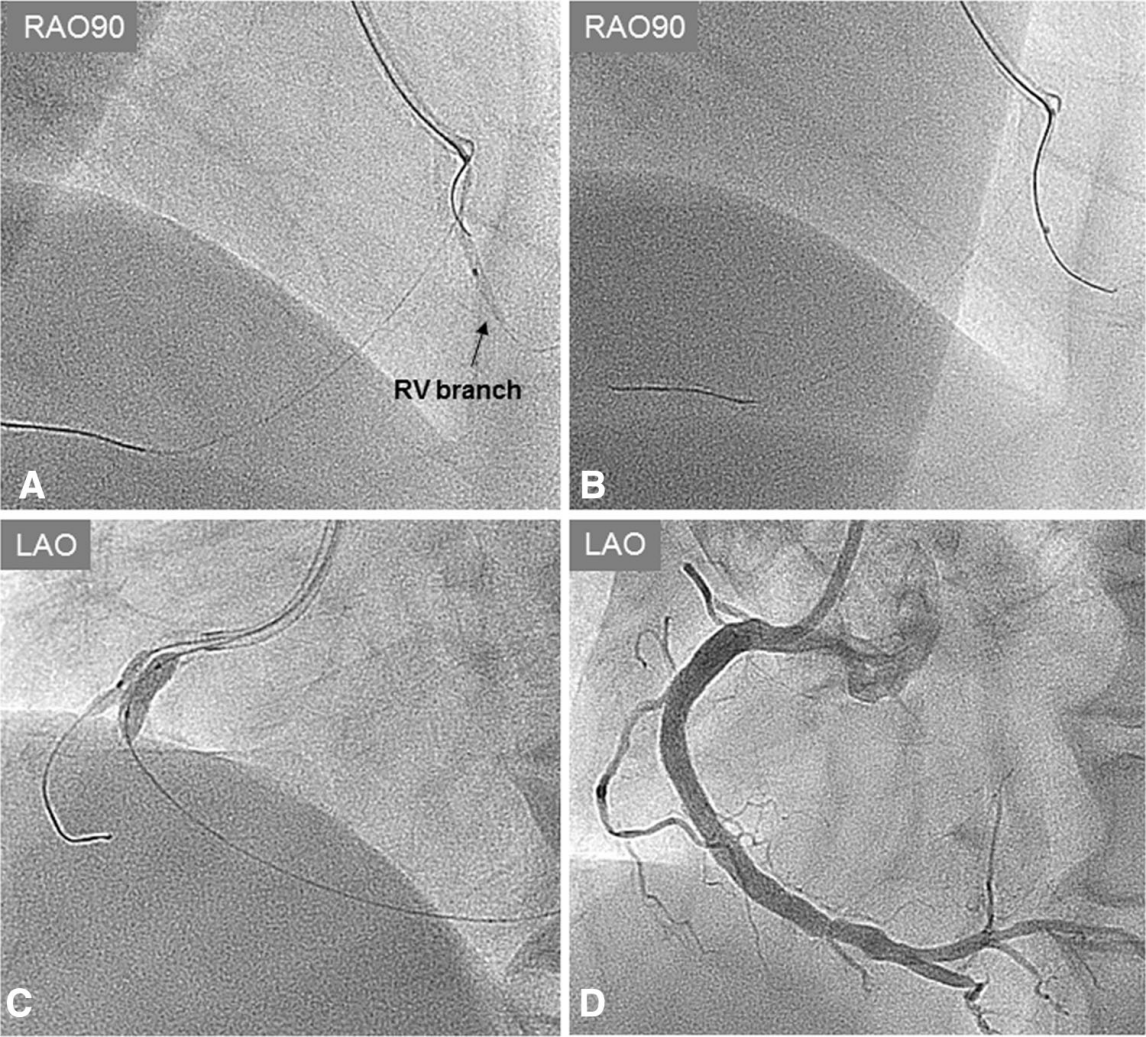
**a** Right anterior oblique (RAO) lateral view. Manipulation of Gaia second guidewire with Crusade catheter during dilation for the right ventricular (RV) branch true lumen by 1.5 mm balloon. **b** While the tip of Gaia second guidewire was facing on the balloon through the strut, immediately balloon deflation was applied. The Gaia second guidewire could pass to the RV branch true lumen in the same space of the first protection guidewire. **c** Kissing balloon inflation at the right coronary artery main vessel and RV branch with 4.0 mm and 2.0 mm balloon, respectively. **d** Final angiogram. The RV branch was restored; the main coronary vessel also showed optimal result

A 58-year-old male was admitted to our hospital because of an acute inferior myocardial infarction. An emergency coronary angiogram revealed the right coronary artery (RCA) total occlusion just distal of RV branch bifurcation. PCI was performed for the culprit lesion. After crossing the guidewire to the RCA distally and to the RV branch, we used an intravascular ultrasound (IVUS) for examination following a thrombectomy by aspiration catheter. IVUS showed attenuated plaque at the lesion site involving the RV branch orifice (Fig. 1). After a 2.0 mm balloon dilation for the culprit lesion, a filter wire (Filtrap; Nipro, Japan) was advanced into the distal RCA to reduce distal embolization and 4.0 × 28 mm Liberte stent (Boston scientific, MA) was implanted at 10 atm. After stenting, the patient’s chest pain worsened and electrocardiography showed an ST segment re-elevation in leads II, III, and aVf. An angiogram revealed a filter block. The Filtrap was removed and injected with nicorandil, and the main coronary flow was improved. However, the patient continued to experience chest pain and ST elevation in leads from V1 to V3. The angiogram showed that the RV branch was completely occluded (Fig. 2). Because the occluded RV branch was the only major RV branch and an RV infarction might have occured if the RV branch was lost, we attempted rewiring to the RV branch using ASAHI SION blue guidewire (ASAHI Intecc, Japan) with a Crusade catheter (Kaneka, Japan). The second guidewire entered into the SB but the tip did not go distally enough that the second guidewire got into the subintimal space of the RV branch. Although we tried to recross several times, the results were the same. IVUS image at the RV branch bifurcation from the RCA MV revealed a jailed first guidewire in the true lumen that was collapsed by an expanded false lumen presented as a high echogenic lumen (Fig. 2). The RV orifice was also sealed by shifted plaque. In this situation, the second guidewire was likely to enter into the expanded false lumen, and it was impossible to recross into the collapsed true lumen. In order to pick up the true lumen, enlarging the true lumen was a key point. We decided to dilate the RV branch behind the stent strut and pick it up from inside the stent (Fig. 3). A 1.5 mm balloon could not enter the vessel behind the deployed stent struts. First, a Corsair 135 cm catheter (ASAHI Intecc, Japan) was advanced into the RV branch on the protection jailed guidewire behind the stent struts. The Corsair catheter successfully reached the RV branch. Then, we removed the Corsair catheter and inserted 1.5 mm balloon on the same guidewire. While the RV branch balloon dilation was in the true lumen, we aimed the RV branch balloon with the ASAHI Gaia second guidewire, supported by Crusade catheter, on the MV guidewire (Fig. 4a). While the ASAHI Gaia second guidewire tip was pushing on the balloon through the strut, the balloon was immediately deflated. The wire picked up the RV true lumen in the same space of the first protection guidewire without hard resistance (Fig. 4b). The ASAHI Gaia second guidewire was advanced more distally, and the Crusade catheter was removed. The Corsair catheter was advanced on the Gaia second guidewire and confirmed in the true lumen by tip injection from the Corsair catheter. To avoid vessel injury or wire perforation, we changed to a floppy wire through the Corsair catheter and then pulled out the Corsair catheter. IVUS image of the RV branch showed that the recross route was in the true lumen and the false lumen was collapsed. Kissing balloon inflation was performed with 4.0 mm and 2.0 mm balloon for the RCA stent and the RV branch, respectively (Fig. 4c). A final angiogram showed the recanalized RV branch and optimal dilation of the RCA MV (Fig. 4d). ST elevation in leads from V1 to V3 was improved, and the patient’s symptoms were resolved.

## Discussion

An SB abrupt occlusion after MV stent implantation is sometimes complicated in the bifurcation PCI. Hahn JY et al. reported that the presence of a stenotic lesion at the SB and proximal MV are independent predictors of SB occlusion after MV stenting [2]. In our case, the lesion had both findings and was considered at high risk for SB occlusion.

To avoid an SB occlusion, we usually insert a protection guidewire into the SB before MV stenting. If SB flow disappears after MV stenting, we can recross the SB using jailed wire as a marker. However, in 3–8 % of bifurcation PCI, occluded SB could not be recanalized [4, 5].

A previous report described the usefulness of an inverted crush technique, particularly if the vessel occlusion causes severe hemodynamic impairment [6, 7]. When the SB is a large vessel like circumflex artery, an inverted crush technique is one of the bailout techniques. However, in this case, the SB was an RV branch that was too small for stent implantation.

Small balloon inflation over a jailed guidewire as a bailout technique in a case of abrupt SB occlusion during provisional stenting was previously reported [8]. When the SB occlusion is caused by plaque shift, this procedure is considered to work well. However, in our case, the SB occlusion was caused not only by plaque shift but also by dissection. In this case, the RV branch true lumen collapsed from an ostium because of dissection. A guidewire could cross through the stent strut but it went always into the false lumen, and it was impossible to pick up the true lumen. To enlarge the ostial true lumen of the RV branch, we dilated the ostial RV branch true lumen with a balloon behind the stent strut. We then aimed the balloon from inside the stent to pick up the RV branch true lumen and succeeded. A recent chronic total occlusion (CTO) procedure in the contemporary reverse controlled antegrade and retrograde subintimal tracking (CART) technique, we aim an antegrade balloon by a Gaia guidewire in retrograde position. The key point of this procedure is pushing the guidewire tip against the balloon. Quick balloon deflation makes it easy to penetrate the tissue between the balloon surface and retrograde guidewire. ASAHI Gaia second guidewire has adequate penetration force and torque controllability. Thus, we chose this guidewire to penetrate the plaque-sealed RV branch ostium. The Crusade catheter is also helpful to control the guidewire in a large MV anatomy.

As a limitation, this case is only one experience; thus, we do not know whether this technique can always work for other lesions. However, it is theoretically possible to attempt for other occluded SB although it is not because of a hematoma. If hemodynamics is tolerated, we can try this maneuver also in the left main bifurcation PCI.

## Conclusion

Our case demonstrated that occluded SB could be rescued using bailout technique, even for a complicated SB dissection. In addition, careful second guidewire manipulation is necessary to avoid creating a new dissection in the SB when we attempt guidewire recrossing to occluded SB after MV stenting.
